# Global scoring method of Ki67 immunohistochemistry in breast cancer demonstrates improved concordance using real-world multi-institutional data

**DOI:** 10.1186/s13058-025-02114-6

**Published:** 2025-09-02

**Authors:** Ceren Boyaci, Wenwen Sun, Johan Hartman, Balázs Ács

**Affiliations:** 1 Department of Oncology and Pathology, Karolinska Institutet, Stockholm, Sweden; 2 Department of Clinical Pathology and Cancer Diagnostics, Karolinska University Hospital, Stockholm, Sweden

**Keywords:** Ki67, Breast cancer, Global scoring, Interobserver variability, IKWG

## Abstract

Ki67 is a broadly available biomarker of proliferation with various approaches to its evaluation in breast cancer. The International Ki67 in Breast Cancer Working Group (IKWG) recommends calculating Ki67 globally across the tumor area, as this method offers high interobserver concordance. These recommendations have been integrated into many international breast cancer guidelines (ASCO, ESMO), yet there is no real-world data on if it improved inter-pathologists and inter-laboratory variability. Here, we present the first real-world data on the variability and impact of Ki67 scores on clinical decision-making in breast cancer when using hotspot scoring and after implementing IKWG global scoring method in clinical practice. We extracted hotspot and global scoring results from 4313 breast cancer patients from routine diagnostics, categorizing the scores into three clinical risk groups over a one-year period across two large breast centers in Stockholm, Sweden. Variability between pathologists was not clinically significant. The global scoring method improved inter-laboratory consistency but also led to a notable increase in cases classified as intermediate at both centers This has been captured in the 2022 Swedish clinical breast cancer guidelines recommending molecular profiling test for patients with Grade II, global Ki67 intermediate score, T1c / T2 breast cancer. To conclude, our findings demonstrate acceptable consistency in Ki67 scores among individual pathologists across both scoring methods, however high inter-laboratory variability was observed with hot spot scoring. Global Ki67 scoring demonstrated low inter-laboratory variability in real world breast cancer care, thereby increasing the reliability of Ki67 assessment in clinical decision-making.

**Supplementary Information:**

The online version contains supplementary material available at 10.1186/s13058-025-02114-6.

## Background

Ki67 is a widely accessible immunohistochemical marker for measuring proliferation in various malignancies, based on the premise that tumors with higher proliferation rates are more aggressive. In breast cancer, Ki67 has been extensively studied for its prognostic and promising predictive value, and has been shown to correlate strongly with Prosigna test scores [1–5]. According to the ESMO Clinical Practice Guideline for early breast cancer, Ki67 is recommended in the pretreatment pathological evaluation [6].

Traditionally, Ki67 staining has been scored according to the “hotspot” approach that focuses on the most proliferative region in invasive breast cancer. However, there are uncertainties regarding whether tumor behavior is accurately represented by this small area, potentially leading to overestimating of aggressiveness, although some researchers claimed the opposite [7]. Other limitations of the hotspot scoring method include intra- and inter-observer variability due to intra-tumoral heterogeneity and difficulty in identifying precisely the “hotspot” region due to lack of well-defined criteria [8, 9].

The International Ki67 Working Group (IKWG) has put great effort to standardize and improve Ki67 assessment aiming to achieve analytical validity and enhance its clinical utility. They reached high interobserver concordance using global scoring method in several rounds of international round-robin setting (multiple pathologists independently evaluating the same set of slides) in a cohort of 30 primary estrogen receptor positive breast cancers [8]. IKWG has suggested that the results that are below 5% (Ki67 low) or above 30% (Ki67 high) can be used in clinical setting to determine the patient group that may benefit from adjuvant chemotherapy [10].

While global scoring can be time-consuming in comparison to hotspot scoring, it offers a better representation of tumor heterogeneity [8]. Although AI-based digital pathology tools that facilitate global scoring are available (e.g. Visiopharm, Mindpeak, Aiforia), their adoption remains limited due to varying levels of accessibility worldwide, mainly driven by cost, validation requirements, and regulatory barriers [11].

In Sweden, Ki67 is routinely included in breast cancer panels for pathological assessment and is used to divide ER-positive, HER2-negative tumors in clinical risk categories [12]. According to the recommendation from the Quality and Standardization Committee of the Swedish Association for Pathology (KVAST), the “weighted global score” method by the IKWG was implemented on March 1, 2022, for both biopsy and resection specimens in cases of invasive breast cancer [8, 13, 14]. Prior to that, hotspot scoring was used [15]. The main reason of this transition was to improve reproducibility and reduce variability of Ki67 between pathologists [15].

In this study, we aimed to report the first real-world data on implementing IKWG Ki67 global scoring in breast cancer patients from two large breast center hospitals in Stockholm, Sweden. The variability and impact of global scoring on clinical decision-making recommendation was compared to that of hot spot scoring which was the standard method before implementing IKWG guidelines.

## Materials and methods

### Study population and data retrieval

Before March 1, 2022, hotspot scoring was used to assess Ki67 in routine practice in breast pathology. A three-month transition period was applied after that date, as it took time for pathologists to adapt the new scoring method following the introduction of new guidelines suggesting the global score.

We retrieved all invasive breast cancer cases with available Ki67 scores diagnosed between June 1, 2021, and May 31, 2022 (Period 1), as well as those diagnosed between June 1, 2022, and May 31, 2023 (Period 2) at two pathology clinics of Karolinska University Hospital (Södersjukhuset [SH] and Solna) from the laboratory information system. The hotspot scores that were given during the routine diagnostic process were registered for Period 1 and the global scores were registered for Period 2. If multiple Ki67 scores were available for patients with multifocal disease, all Ki67 scores were recorded. A total of 2544 patients from SH and 1769 from Solna were included in the study.

### Immunohistochemical Ki67 staining

The immunohistochemical staining of Ki67 with 30 − 9 clone (Roche diagnostics, Ventana, USA) was applied on all invasive breast cancer cases as a part of routine diagnostic work in Ventana Benchmark auto stainer. Both clinics use the same assay and stainer.

### Hotspot scoring

Hotspot scoring was done on glass slides using light microscope on x40 magnification and performed in the tumor area with the highest density of Ki67 stained invasive cancer cells. The calculation was based on a minimum of 200 tumor cells.

### Weighted global scoring

Slides were digitized at 40x magnification using Hamamatsu scanners (Hamamatsu Photonics, Japan) as part of the routine diagnostic workflow. Weighted global scoring was performed as described by IKWG [16]. Shortly, analysis focused on invasive tumor cells, excluding in situ regions. Areas with varying levels of Ki67 expression and their respective percentages were identified. A total of 400 cells were counted, with 100 cells analyzed from four selected fields of view, each representing different Ki67 expression areas [13]. Ki67 scoring tool (Sectra AB, Linköping, Sweden), which had been integrated into the laboratory information management system, facilitated the scoring. This tool automatically calculates a predefined number of cells within a region of interest selected by pathologist; however, it also allows correction by pathologist operator.

**Fig. 1:**
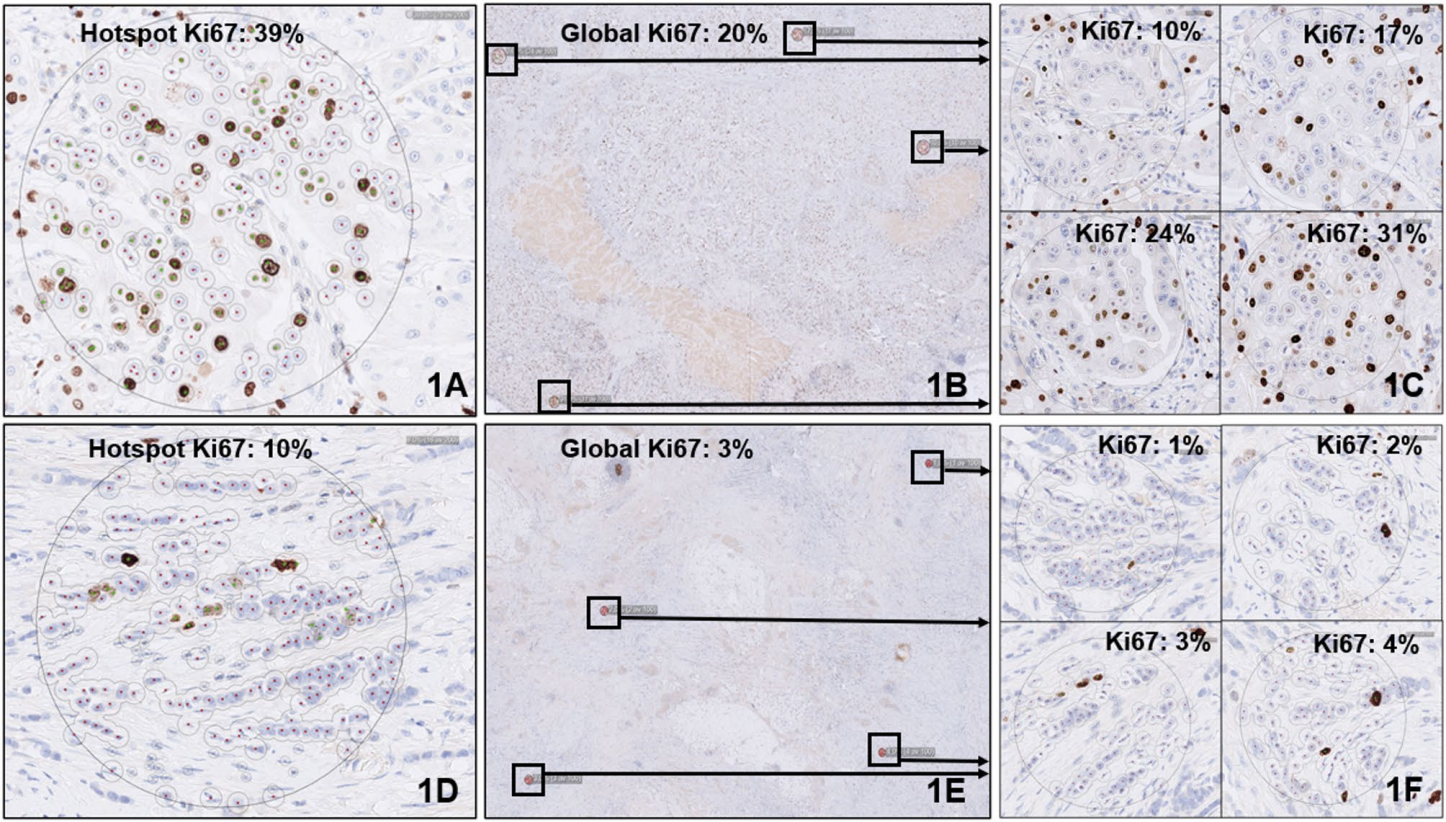
Case 1 exhibits heterogeneous Ki67 staining. The hotspot Ki67 index is 39%, which falls into the high proliferation category (Fig. 1A). However, the global score, which is calculated on four regions (10%, 17%, 24%, and 31%) to capture Ki67 expression heterogeneity, is 20%, placing it in the intermediate group (Fig. 1B). Case 2 has a hotspot Ki67 index of 10%, placing it in the low proliferation group (Fig. 1D). The global score, calculated across four regions (1%, 2%, 3%, and 4%), yields an average of 3%, which also classifies it within the low group (Fig. 1E). Figure 1C and F show four high-power field regions that were included in the global scoring for each respective case. In each field, the software counted 100 tumor nuclei within the circular area

Two examples of cases with different Ki67 staining patterns are presented in Fig. 1 demonstrating the scoring methods.

### Ki67 cut-offs

For hotspot scoring, Ki67 intervals were defined separately for each center based on the median values as follows: Low (0–14%), intermediate (15–22%), and high (23–100%) for Solna and low (0–14%), intermediate (15–24%), and high (25–100%) for SH [17, 18]. For global scoring, the intervals were redefined as: Low (< 6%), intermediate (6–29%), and high (> 29%) according to suggestions of IKWG and KVAST, same for both centers [12].

### Pathologists

Pathologists at SH coded as P1-P16 (*n* = 16) and pathologists at Solna as PA-PG (*n* = 7). Pathologists who diagnosed minimum 40 breast cancer patients and scored minimum 30 cases per category (hotspot biopsy/global biopsy/hotspot resection/global resection) were included in the statistical analysis for the assessment of interobserver concordance.

### Statistical analysis

SPSS 27 software was used for statistical analysis (IBM, Armonk, NY, USA). Kruskal-Wallis test was applied when comparing Ki67 scores among pathologists. All pathologists within the same department were compared pairwise, resulting in a total of 28 comparisons per department. Significance values were adjusted by Bonferroni correction for multiple tests. Additionally, Chi-square test was applied to compare distribution of Ki67 categorical scores separately in hotspot and global method (Table 1).

## Results

### Clinicopathological characteristics

**Table 1:** Multiple Ki67 scores were available for patients with multifocal disease. IQR: interquartile range

	SH / Hotspot	Solna / Hotspot	SH / Global	Solna / Global
Total biopsies	658	390	700	533
Total resections	557	330	635	597
Total patients	1247	688	1297	1081
Median age (range)	66 (21–99)	65 (24–96)	64 (30–97)	65 (28–97)
Median Ki67, IQR	25 (15–43)	19 (10–32)	19 (11–35)	17 (8–30)

A total of 1247 respectively 688 patients were evaluated using the hotspot method for SH and Solna while 1297 respectively 1081 patients were assessed with the global method. The median age was relatively consistent across groups, ranging between 64 and 66 years. With the hotspot method, median Ki67 was higher in both centers (Table 1).

The number of pathologists that met the predefined criteria of scoring minimum 30 cases per category (hotspot biopsy/global biopsy/hotspot resection/global resection) were 7/8/7/5 for SH and 7/5/5/5 for Solna, respectively.

### Variability of Ki67 scoring methods in biopsy specimens

We found no statistically significant differences in hotspot Ki67 scores between pathologists in both clinics for biopsy specimens (*p* = .218 and *p* = .154) (Fig. 2A).

**Fig. 2 A:**
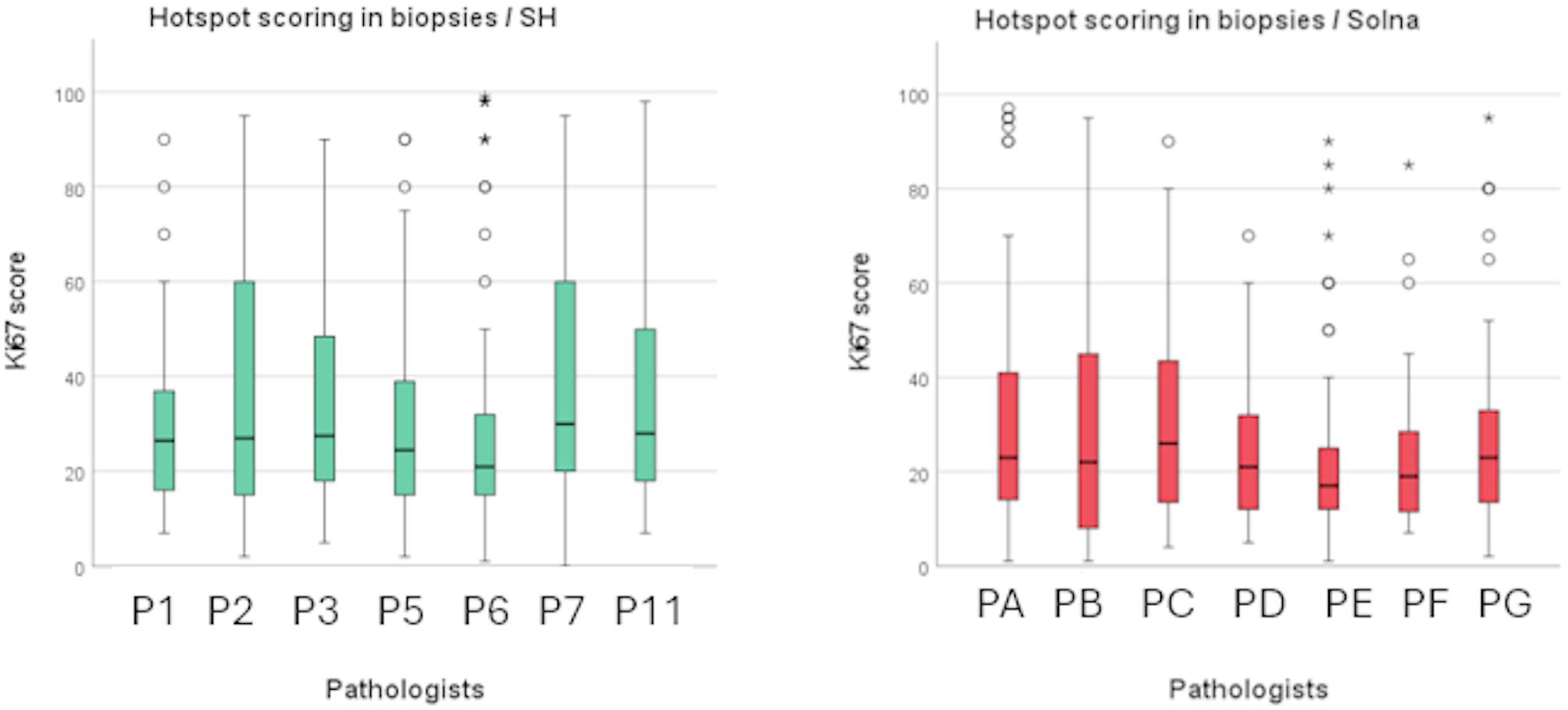
Boxplots showing the distribution of Ki67 scores in biopsy specimens across different pathologists for SH and Solna using hotspot method

A statistically significant difference was found in one of 28 comparisons between pathologists (P4 vs. P6) in global Ki67 scores for SH (*p* = .001). For Solna, no statistically significant differences were observed in global Ki67 scores between pathologists for biopsy specimens (*p* = .374) (Fig. 2B).

**Fig. 2B:**
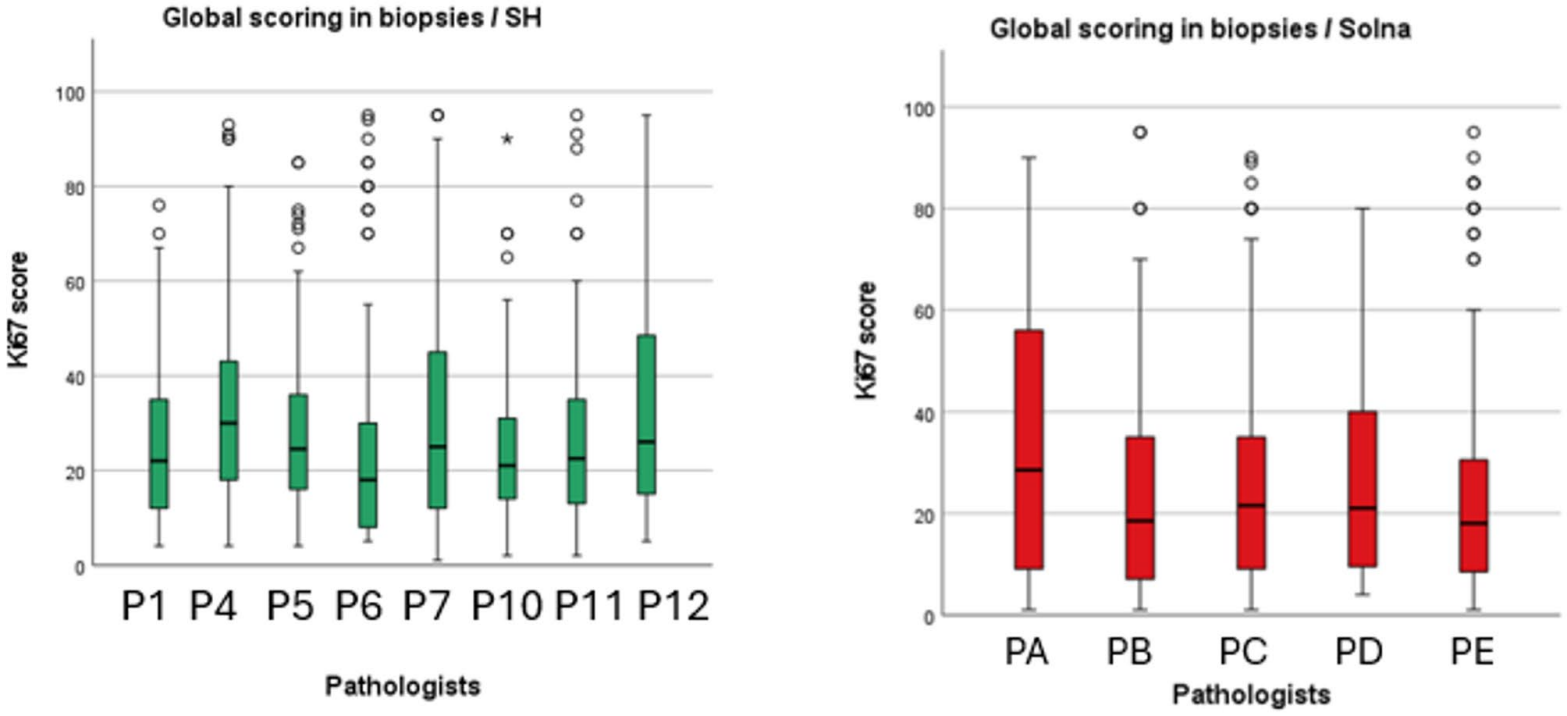
Boxplots showing the distribution of Ki67 scores in biopsy specimens across different pathologists for SH and Solna using the global method

### Variability of Ki67 scoring methods in resection specimens

We found no statistically significant differences in hotspot and global Ki67 scores between pathologists in both clinics for resection specimens (*p* = .637 and *p* = .064) (*p* = .68 and *p* = .104) (Fig. 3A and B).

Additionally, Ki67 scoring results from pathologists at SH tend to have a median value above 20%, while Solna scores are typically lower for hotspot scoring in resections (Fig. 3A). In contrast, with global scoring, the score distribution becomes similar between SH and Solna, indicating an increased consistency across departments (Fig. 3B).

**Fig. 3 A:**
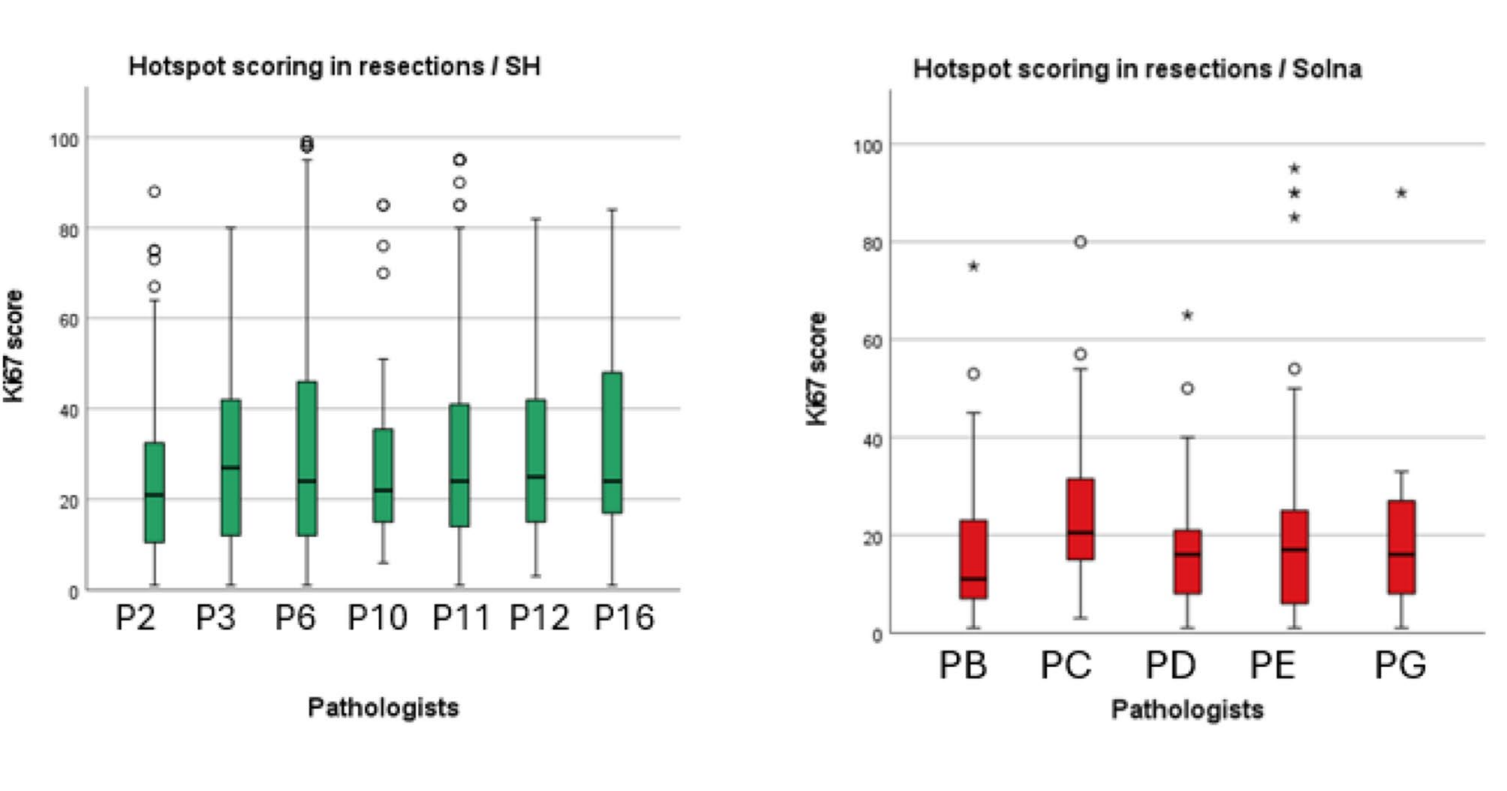
Boxplots showing the distribution of Ki67 scores in resection specimens across different pathologists for SH and Solna using the hotspot method

**Fig. 3B:**
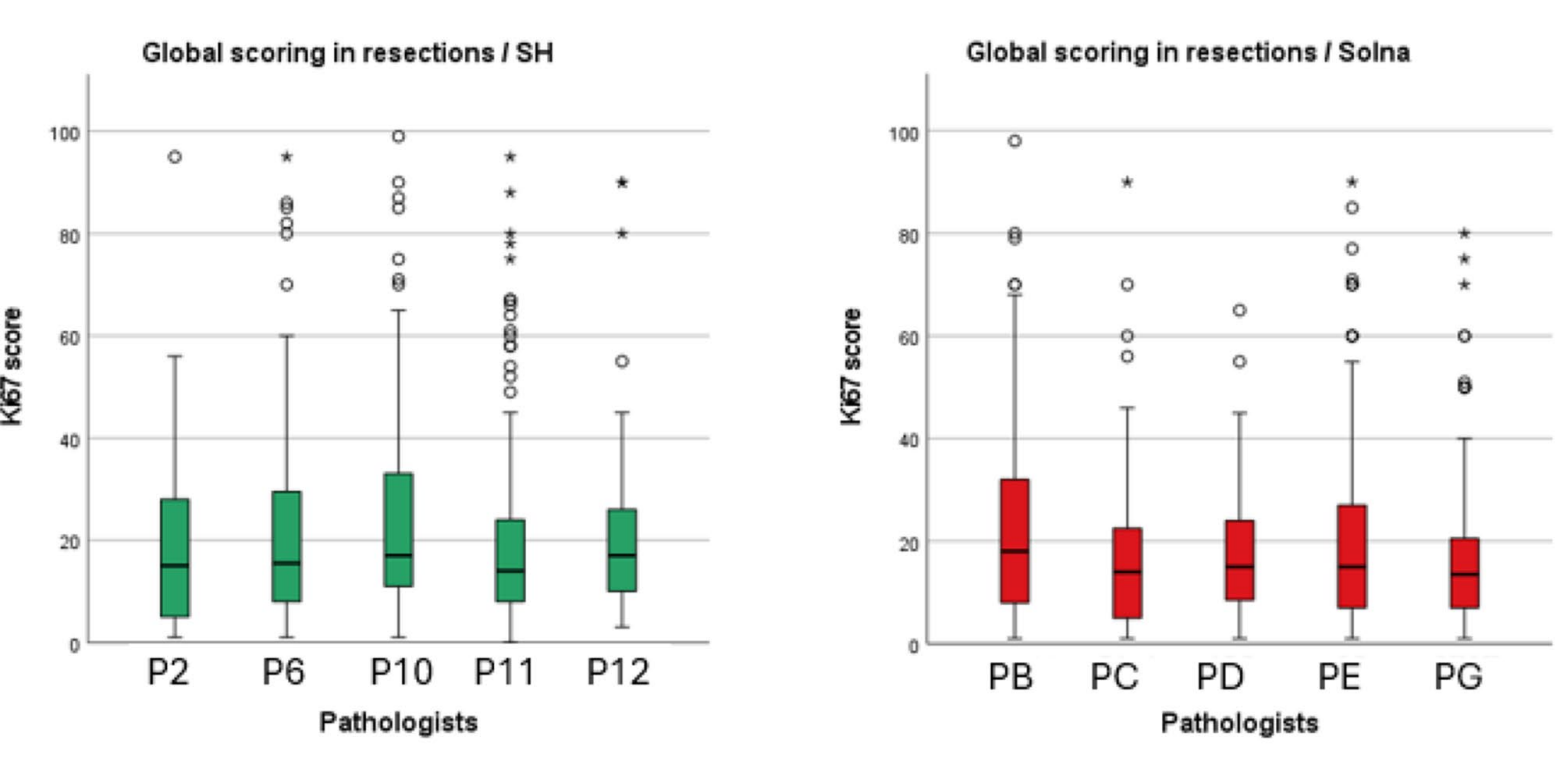
Boxplots showing the distribution of Ki67 scores in resection specimens across different pathologists for SH and Solna using the global method

### Impact of Ki67 on clinical decision-making recommendation

**Fig. 4:**
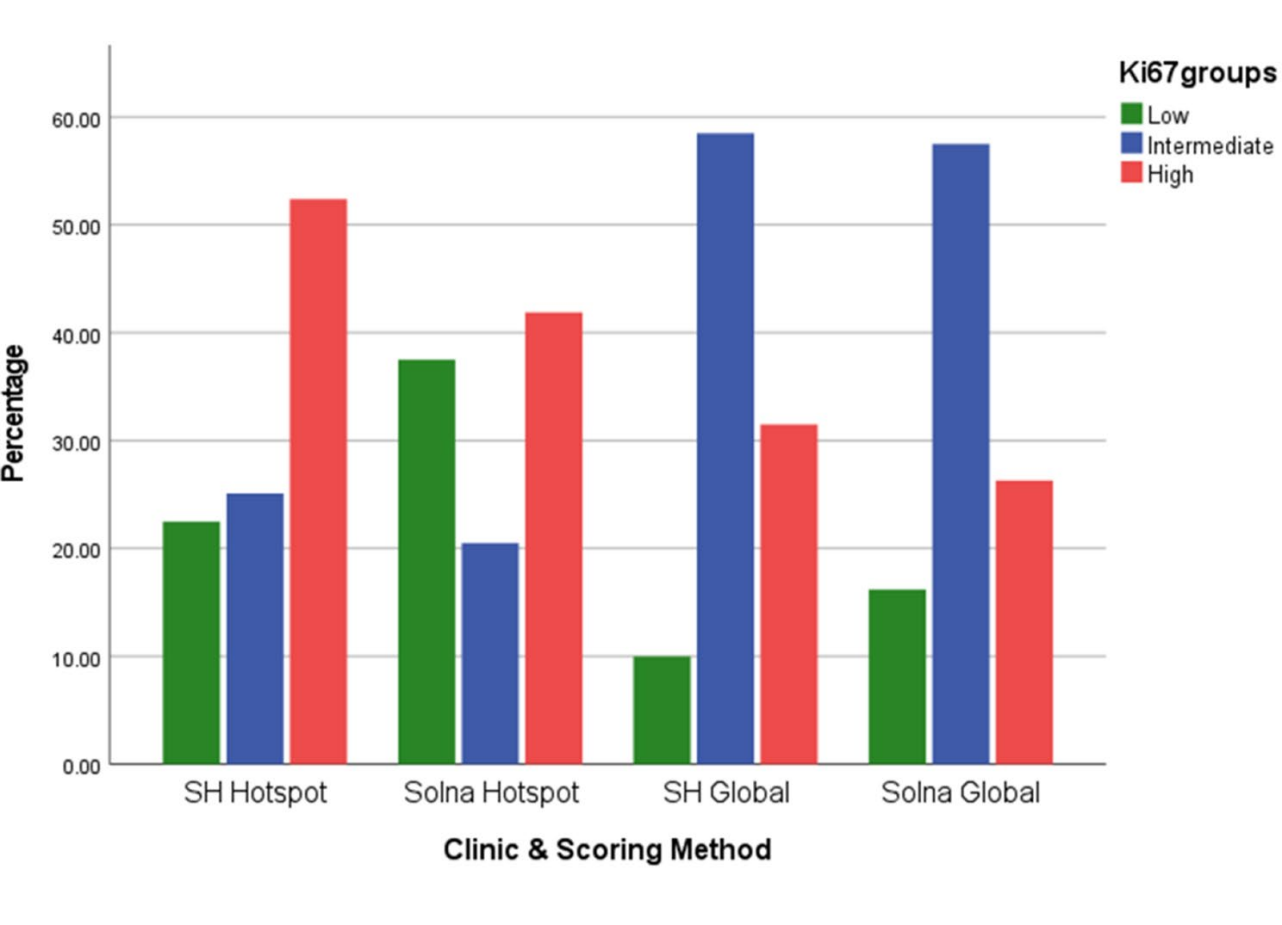
The bar chart displays the distribution of Ki67 risk categories over two periods at two clinics—SH and Solna—employing two different scoring methods

In clinical practice, Ki67 is used as categorical variable: low, intermediate and high. We observed that a greater proportion of cases (52.4% and 41.9%) fell in the high category using the hotspot method. The global method resulted in a significant increase in cases classified as intermediate, rising from 25,1% to 58,5% in SH and from 20,5% to 57,5% in Solna. Using the hotspot method, the distribution of cases across Ki67 categories varied between the clinics (Fig. 4). On the contrary, a more similar distribution (but statistically significant difference (*p* < .001)) was seen between SH and Solna with the global method, almost 60% of cases in both clinics fell into the intermediate category (Fig. 4). The same distribution of Ki67 groups was observed when biopsy and resection specimens evaluated separately (Supplementary 1 A-B). The higher rate of Ki67 intermediate category by the global method has been interpreted in the 2022 Swedish clinical decision tree recommending gene expression profiling for patients with Grade II, Ki67 intermediate, T1c / T2 breast cancer (Supplementary 3).

## Discussion

Ki67 is a simple and valuable tool in breast cancer management, whereas its interpretation can vary due to differences in scoring methods, such as hotspot and global scoring. Standardization efforts by IKWG aimed to improve the scoring consistency and to enhance clinical utility of Ki67 in routine practice resulting in global scoring is recommended by both ASCO and ESMO international breast cancer guidelines.

Therefore, we investigated the variability of Ki67 scores using two different scoring methods in routine practice across two clinics and explored how the transition from hotspot to global scoring has impacted clinical decision-making guidelines. To our knowledge, this is the first study that investigates the recommendations of IKWG and the variability of different scoring methods in real-world data, however there are previous studies focusing on the analytical validity of Ki67 in breast cancer [8, 19].

Before starting this study, we hypothesized that variability between pathologists would be higher with the hotspot scoring due to subjective nature of the method [20]. Still, we found acceptable consistency between individual pathologists regardless of the scoring method.

Our findings suggest that variability between individual pathologists is not as pronounced as expected, even in resection specimens that encompass a larger area than biopsies. Standardization of pre-analytical variables, even though cases were stained at two laboratories and evaluated by different pathologists working at those centers, may have contributed to this result. Furthermore, our findings suggest that pathologists at the same department have the same scoring tendencies.

In the current study, while the pathologists’ scores are consistent with those of their colleagues with both scoring methods, a significant variability between laboratories exists. This finding aligns with the results from our earlier study, which observed inter- and intra-laboratory variability in pathological parameters, including Ki67, within a national cohort in Sweden [15]. After transitioning to global scoring, the distribution of Ki67 categories shifted significantly, with an increase in the number of cases categorized as intermediate, resulting in a more similar distribution between the centers. For this reason, global scoring is recommended for better alignment across centers.

While our study highlights notable differences in Ki67 risk categories depending on the method used, not all studies have reported such discrepancies. For instance, in the study of Jang et al., no significant differences were observed in the categorization of 493 tumors (Ki67 low vs. high) when comparing hotspot and global scoring methods [20]. However, their study is not directly comparable with this current study due to differences in the definition of global scoring, the cut-off values applied, and patient population involved.

From a biological perspective, global scoring provides a more accurate reflection of the overall tumor proliferation status. Zilenaite-Petrulaitiene et al. observed lower intratumoral heterogeneity in cases at both ends of the Ki67 proliferation index scale when using a study-specific cut-off value [21]. Moreover, establishing a universal cut-off for dichotomization is challenging for a continuous marker as Ki67, because it may not apply consistently across pathology departments due to pre-analytic and analytic variability. Therefore, defining an intermediate category with the proposed cut-off values from the IKWG can be valuable, as it may identify a subgroup with higher intratumoral heterogeneity and uncertain behavior, highlighting the need for additional information about tumor biology, potentially supported by multigene tests or AI-based analysis [22, 23].

Half of the studied breast cancers fall into the intermediate grade category that provides limited clinical value for guiding patient management [15, 24]. Furthermore, the expansion of the intermediate Ki67 group may present a new challenge: an increased number of cases with uncertain treatment decisions, which may lead to greater reliance on molecular testing to guide adjuvant therapy. This raises concerns about fair access to health care, as patients who cannot get these tests may have less treatment options. On the other hand, high variability in Ki67 scoring between pathology labs can also cause inequality in cancer care. To ease these concerns, other established clinicopathologic factors (e.g.: age, tumor size, grade, lymph node status, hormone receptor status) considered together with Ki67 may aid the decision-making.

Ki67 scores are clinically relevant particularly for early-stage luminal/HER2-negative breast cancer in post-menopausal patients, whereas treatment choices for triple-negative and HER2-positive cancers are mostly independent of Ki67 [25]. In the latest St. Gallen Conference, Ki67 and change of Ki67 by treatment were mentioned as prognostic biomarkers with limited value in influencing adjuvant chemotherapy decisions [26]. Ki67 is also a companion diagnostic for the FDA-approved abemaciclib therapy in the USA, specifically for patients with a Ki67 score of ≥ 20% measured by a specific FDA-approved test. However, there are criticisms regarding the predictive value of this cut-off in real-world scenarios, particularly concerning the sparse evidence on reproducibility [27, 28]. In Stockholm, the clinical guidelines changed in 2022 and global Ki67 has been incorporated together with grade, tumor size, ER/PR/HER2 status in the decision-making on molecular profiling test and or on adjuvant chemotherapy. Molecular profiling test is recommended for patients with T1c/T2, grade 2, Ki67 intermediate and ER positive/Her2 negative breast cancer. For all the other ER positive/Her2 negative breast cancer patients, the above-mentioned clinicopathologic factors with global Ki67 score shall be used to decide on recommending adjuvant chemotherapy. This context highlights the clinical relevance of refining and standardizing Ki67 scoring methods.

As a limitation of our study, we did not account for the clinical- and molecular subtypes of breast cancer. Furthermore, our study was not prospectively designed to demonstrate clinical utility. However, only randomized clinical trials can provide evidence on clinical utility and the international guidelines already recommend assessing Ki67 in clinical practice. Therefore, we believe that our study offers valuable real-world data about the analytical validity of implementing global Ki67 scoring in clinical routine.

## Conclusion

In conclusion, we demonstrated no significant variability of Ki67 scores between individual pathologists, while global scoring has resulted in a similar distribution in Ki67 risk categories. On the other hand, hot spot scoring showed high inter-laboratory variability. This highlights the importance of implementing the IKWG global Ki67 scoring guidelines to enhance the risk assessment of breast cancer patients in clinical decision-making.

## Electronic Supplementary Material

Below is the link to the electronic supplementary material.

Supplementary Material 1

## Data Availability

No datasets were generated or analysed during the current study.

## Abbreviations

IKWG: The International Ki67 in Breast Cancer Working Group
ASCO: American Society of Clinical Oncology
ESMO: European Society for Medical Oncology
ER: Estrogen receptor
PR: Progesterone receptor
HER2: Human epidermal growth factor receptor 2
KVAST: The Quality and Standardization Committee of the Swedish Association for Pathology
SH: Södersjukhuset
FDA: The Food and Drug Administration
NHG: Nottingham Histologic Grade

## References

[1] Davey MG, Hynes SO, Kerin MJ, Miller N, Lowery AJ. Ki-67 as a prognostic biomarker in invasive breast cancer. Cancers (Basel). 2021. 10.3390/cancers13174455. 34503265 10.3390/cancers13174455 PMC8430879

[2] Gown AM. The biomarker Ki-67: promise, potential, and problems in breast cancer. Appl Immunohistochem Mol Morphol. 2023. 10.1097/PAI.0000000000001087. 36730064 10.1097/PAI.0000000000001087

[3] Petrelli F, Viale G, Cabiddu M, Barni S. Prognostic value of different cut-off levels of Ki-67 in breast cancer: a systematic review and meta-analysis of 64,196 patients. Breast Cancer Res Treat. 2015. 10.1007/s10549-015-3559-0. 26341751 10.1007/s10549-015-3559-0

[4] Baskota SU, Dabbs DJ, Clark BZ, Bhargava R. Prosigna^®^ breast cancer assay: histopathologic correlation, development, and assessment of size, nodal status, Ki-67 (SiNKTM) index for breast cancer prognosis. Mod Pathol. 2021. 10.1038/s41379-020-0643-8. 32740650 10.1038/s41379-020-0643-8

[5] Loibl S, André F, Bachlot T et al. Early breast cancer: ESMO clinical practice guideline for diagnosis, treatment and follow-up. Ann. Oncol. 2024;35:159–182. 10.1016/j.annonc.2023.11.016 10.1016/j.annonc.2023.11.016 38101773

[6] Mu K, Li L, Yang Q, et al. A standardized method for quantifying proliferation by Ki-67 and Cyclin A immunohistochemistry in breast cancer. Ann Diagn Pathol. 2015. 10.1111/his.14781. 26049669 10.1016/j.anndiagpath.2015.05.002

[7] Leung SCY, Nielsen TO, Zabaglo LA, et al. Analytical validation of a standardised scoring protocol for Ki67 immunohistochemistry on breast cancer excision whole sections: an international multicentre collaboration. Histopathology. 2019. 10.1111/his.13880. 31017314 10.1111/his.13880

[8] Robertson S, Acs B, Lippert M, Hartman J. Prognostic potential of automated Ki67 evaluation in breast cancer: different hot spot definitions versus true global score. Breast Cancer Res Treat. 2020. 10.1007/s10549-020-05752-w. 32572716 10.1007/s10549-020-05752-w PMC7376512

[9] Nielsen TO, Leung SCY, McShane LM, Dowsett M, Hayes DF. Response to Zhang and Yang. J Natl Cancer Inst. 2021. 10.1038/s41379-022-01104-9. 34003287 10.1093/jnci/djab094 PMC8562955

[10] National care program breast cancer. 2024. https://kunskapsbanken.cancercentrum.se/diagnoser/brostcancer/vardprogram/. Accessed 10 November 2024.

[11] Swedish Society of Pathology—Quality and Standardization Committee (KVAST): Breast Cancer Guideline. 2024. https://svfp.se/kvast/brostpatologi/kvast-dokument/. Accessed 10 November 2024.

[12] Nielsen TO, Leung SCY, Rimm DL, et al. Assessment of Ki67 in breast cancer: updated recommendations from the international Ki67 in breast cancer working group. J Natl Cancer Inst. 2021. 10.1093/jnci/djaa201. 33369635 10.1093/jnci/djaa201 PMC8487652

[13] Acs B, Frederiksson I, Rönnlund C, et al. Variability in breast cancer biomarker assessment and the effect on oncological treatment decisions: A nationwide 5-year population-based study. Cancers (Basel). 2021. 10.3390/cancers13051166. 33803148 10.3390/cancers13051166 PMC7963154

[14] Leung SCY, Nielsen TO, Zabaglo L, et al. Analytical validation of a standardized scoring protocol for Ki67: phase 3 of an international multicenter collaboration. Npj Breast Cancer. 2016. 10.1038/npjbcancer.2016.14. 28721378 10.1038/npjbcancer.2016.14 PMC5515324

[15] Swedish Society of Pathology—Quality and Standardization Committee (KVAST): Breast Cancer Guideline. 2018. https://svfp.se/media/ejqfcth1/kvastbrostcancer2018.pdf. Accessed 10 November 2024.

[16] Coates AS, Winer EP, Goldhirsch A, et al. Tailoring therapies-improving the management of early breast cancer: St Gallen international expert consensus on the primary therapy of early breast cancer 2015. Ann Oncol. 2015. 10.1093/annonc/mdv221. 25939896 10.1093/annonc/mdv221 PMC4511219

[17] Jang MH, Kim HJ, Chung YR, Lee Y, Park SY. A comparison of Ki-67 counting methods in luminal breast cancer: the average method vs. the hot spot method. PLoS ONE. 2017. 10.1371/journal.pone.0172031. 28187177 10.1371/journal.pone.0172031 PMC5302792

[18] Zilenaite-Petrulaitiene D, Rasmusson A, Besusparis J, et al. Intratumoral heterogeneity of Ki67 proliferation index outperforms conventional immunohistochemistry prognostic factors in Estrogen receptor-positive HER2-negative breast cancer. Virchows Arch. 2024. 10.1007/s00428-024-03737-4. 38217716 10.1007/s00428-024-03737-4

[19] Sestak I, Buss R, Cuzick J, et al. Comparison of the performance of 6 prognostic signatures for Estrogen receptor–positive breast cancer a secondary analysis of a randomized clinical trial. JAMA Oncol. 2018. 10.1001/jamaoncol.2017.5524. 29450494 10.1001/jamaoncol.2017.5524 PMC5885222

[20] Sharma A, Lövgren SK, Eriksson KL, et al. Validation of an AI-based solution for breast cancer risk stratification using routine digital histopathology images. Breast Cancer Res. 2024. 10.1186/s13058-024-01879-6. 39143539 10.1186/s13058-024-01879-6 PMC11323658

[21] van Dooijeweert C, van Diest P, Willems SM, et al. Significant inter- and intra-laboratory variation in grading of invasive breast cancer: a nation wide study of 33,043 patients in the Netherlands. Int J Cancer. 2020. 10.1002/ijc.32330. 30977119 10.1002/ijc.32330 PMC6916412

[22] Cheang MCU, Chia SK, Voduc C, et al. Ki67 index, HER2 status, and prognosis of patients with luminal B breast cancer. J Natl Cancer Inst. 2009. 10.1093/jnci/djp082. 19436038 10.1093/jnci/djp082 PMC2684553

[23] Goldhirsch A, Wood WC, Coates AS, et al. Strategies for subtypes-dealing with the diversity of breast cancer: highlights of the St Gallen international expert consensus on the primary therapy of early breast cancer 2011. Ann Oncol. 2011. 10.1093/annonc/mdr304. 21709140 10.1093/annonc/mdr304 PMC3144634

[24] Andre F, Ismaila N, Allison KH, et al. Biomarkers for adjuvant endocrine and chemotherapy in early-stage breast cancer: ASCO guideline update. J Clin Oncol. 2022. 10.1200/JCO.22.00069. 35439025 10.1200/JCO.22.00069

[25] Dowsett M, Nielsen TO, Rimm DL, Hayes DF. Ki67 as a companion diagnostic: good or bad news? J Clin Oncol. 2022. 10.1200/JCO.22.00581. 35816627 10.1200/JCO.22.00581

[26] Rimm DL, Dacic S, Schnitt SJ. The pathologists’ conundrum. Arch Pathol Lab Med. 2023. 10.5858/arpa.2022-0226-ED. 36577091 10.5858/arpa.2022-0226-ED

[27] Curigliano G et al. Understanding breast cancer complexity to improve patient outcomes: the St Gallen International Consensus Conference for the Primary Therapy of Individuals with Early Breast Cancer. 2023; 10.1016/j.annonc.2023.08.017 10.1016/j.annonc.2023.08.017 37683978

[28] Rimm DL, Dacic S, Schnitt SJ. The pathologists’ conundrum. Arch Pathol Lab Med. 2023;147:17–18. 10.5858/arpa.2022-0226-ED 36577091

