# Supplementary material for: Global scoring method of Ki67 immunohistochemistry in breast cancer demonstrates improved concordance using real-world multi-institutional data
Source: Breast Cancer Res. 2025 Sep 2;27:159. doi: 10.1186/s13058-025-02114-6 (PMC12406372; doi:10.1186/s13058-025-02114-6)
Supplement: Supplementary file 1 — Supplementary Material 1 [file 13058_2025_2114_MOESM1_ESM.docx]

**Supplementary material**


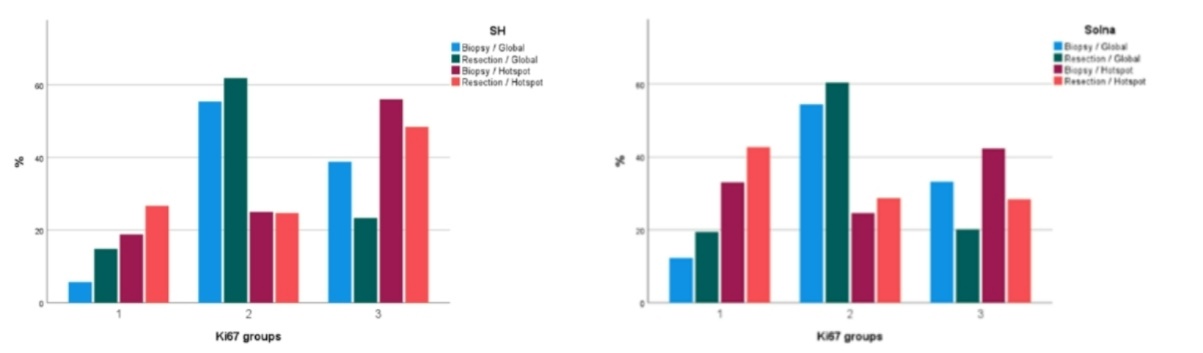


**Supplementary 1A-B:** The bar charts illustrate the distribution of Ki67 categories using two different scoring methods, shown separately for biopsy and resection specimens at SH and Solna.


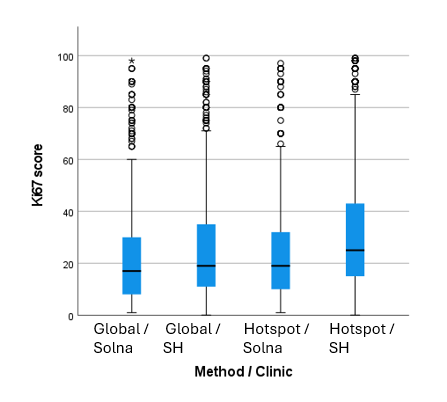


**Supplementary 2:** Boxplots showing the distribution of Ki67 scores over two periods at two clinics—SH and Solna—employing two different scoring methods (Hotspot SH vs. Hotspot Solna: p = .000, Global SH vs. Global Solna: p = .000)

Luminal A-like surrogate subtype: NHG I and hotspot Ki67 low (0–14%), PR > 20%, Luminal B-like surrogate subtype: NHG III or NHG I-II and hotspot Ki67 high (25–100%)

Luminal A-like surrogate subtype: NHG I or II and global Ki67 low (0–5%), Luminal B-like surrogate subtype: NHG III or NHG II and global Ki67 high (30–100%)

**Supplementary 3A-B:** Tables demonstrating Swedish National guidelines from 2019 and 2022 for adjuvant chemotherapy decision in ER-positive breast cancer patients based on Nottingham Histologic Grade (NHG), Ki67 score, PR, and PAM50 analysis.
